# Oscillatory and behavioral indices of cognitive control dysregulation in young adult binge drinkers are influenced by sex differences

**DOI:** 10.1111/acer.70252

**Published:** 2026-02-16

**Authors:** Vanessa F. U. Thomas, Stephen M. Cruz, Siyuan Huang, Ksenija Marinkovic

**Affiliations:** ^1^ Department of Psychology San Diego State University San Diego California USA; ^2^ Department of Biology San Diego State University San Diego California USA; ^3^ Department of Psychology Pennsylvania State University University Park Pennsylvania USA

**Keywords:** binge drinking, event‐related beta, event‐related theta, sex differences, Stroop task

## Abstract

**Background:**

Binge drinking, defined as episodic alcohol intake reaching intoxication, is prevalent among young adults and linked to impaired cognitive control and risk for alcohol use disorder (AUD). Even though executive deficits contribute to addiction, evidence on their alterations in young adult binge drinkers (BDs) as a function of biological sex remains limited. This study examined cognitive and motor aspects of decision making as reflected in event‐related theta and beta oscillations, respectively. The executive neurobehavioral markers were analyzed in conjunction with alcohol‐related and other self‐reported variables and cognitive functions in BDs versus light drinkers (LDs) of both sexes.

**Methods:**

Sixty‐eight participants (34 BDs, 34 LDs, 50% women) completed a modified Stroop task. Electroencephalography (EEG) signals were analyzed in the time‐frequency domain with Morlet wavelets. Behavioral performance and event‐related theta (4–7 Hz) and beta (15–25 Hz) power were compared across levels of conflict, drinking groups, and sex.

**Results:**

BDs exhibited longer reaction times on high‐conflict trials, reflecting compensatory slowing during cognitive interference. Lower conflict‐induced theta power in BDs than in LDs was evident primarily among women. It was associated with greater alcohol intake and it mediated the impact of binge drinking on degraded task performance among BD women. In contrast, event‐related beta desynchronization during response preparation was attenuated and delayed only in BD men, which was partially mediated by impulsivity.

**Conclusions:**

The observed links between binge drinking and dysregulated cognitive and motor control processes were interpreted in regard to sex‐specific neural alterations. Weaker engagement of cognitive control in BD women may be reflected in an impaired capacity to optimize decisions in challenging situations. Beta decrease during response preparation was especially attenuated in more impulsive BD men, which may contribute to their inability to refrain from risky behaviors. These findings highlight the need to consider biological sex in alcohol‐related neurocognitive research.

## Graphical abstract

This EEG study examined cognitive and motor aspects of decision making during a modified Stroop task (A) in young adult binge drinkers (BDs) and light drinkers (LDs). Besides compensatory response slowing to Stroop interference (B), BDs showed lower Stroop‐induced theta power, which mediated the impact of BD on degraded task performance in BD women (C), indicative of impaired cognitive control. Conversely, impulsivity partially mediated an attenuated beta decrease during response preparation in BD men (D), suggesting sex‐specific pathways of vulnerability in BDs.

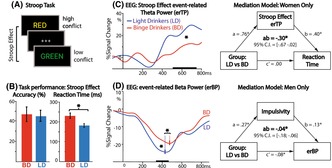

## INTRODUCTION

Binge drinking is prevalent among young adults and is characterized by episodes of heavy alcohol consumption interspersed with periods of no or low‐level drinking. A binge episode is defined as imbibing enough alcohol to reach a blood alcohol concentration (BAC) of at least 0.08 g/dL (National Institute on Alcohol Abuse and Alcoholism, 2016). However, young adults often consume alcohol in greater amounts, which results in much higher BAC levels and enhances potential neurotoxic effects (Patrick & Terry‐McElrath, 2019). Binge drinking interferes with brain maturation, is associated with a range of harms across different functional and organ systems, and with an increased likelihood of engaging in impulsive and risky behaviors (Carbia et al., 2018). Despite these risks and consequences, denial of alcohol‐related problems remains common among emerging adults, and binge drinking is often perceived as nonproblematic and socially acceptable (Bishop & Rodriguez Orjuela, 2018). As a result, this pattern of alcohol misuse might lay the foundation for long‐term consequences, as it increases the risk of developing alcohol use disorder (AUD) (Koob & Volkow, 2010).

Cognitive control is an essential dimension of executive top–down processing that enables flexible behavior in accordance with specific goals, crucial for steering behavior toward or away from tasks. A key aspect of cognitive control is the ability to suppress an automated action in favor of a desired but nonautomated response (Norman & Shallice, 1986). This capacity, together with related functions, such as planning and response inhibition, is part of the executive system, which is often compromised in people with AUD due to prefrontal dysfunction (Le Berre et al., 2017; Oscar‐Berman & Marinković, 2007). Cognitive control deficits make it more difficult for individuals to refrain from risky behavior, including hazardous drinking. Furthermore, they are linked to higher levels of impulsivity, which can contribute to the development and maintenance of AUD (Mitchell & Potenza, 2015). Therefore, influential models propose executive deficits as one of the three core neurofunctional domains critical for addiction (Kwako et al., 2016). Behavioral evidence on impaired cognitive control in binge drinkers (BDs) is scant (Molnar et al., 2018), as large‐scale reviews have failed to find reliable behavioral cognitive deficits except lower inhibitory control in BDs compared with light drinkers (LDs) (Almeida‐Antunes et al., 2021). In contrast, direct measures of neural activity can reliably detect group differences even in small samples in the absence of performance deficits (Almeida‐Antunes et al., 2021; Correas et al., 2019, 2021; Crego et al., 2012; Holcomb et al., 2019; Huang et al., 2022). Extensive evidence indicates that cognitive control is impaired under acute intoxication (Bartholow et al., 2018; Kovacevic et al., 2012; Marinkovic et al., 2019; Marinkovic, Rickenbacher, et al., 2012) as well as in people with AUD (Murray et al., 2022; Oscar‐Berman & Marinković, 2007; Schulte et al., 2012; Wilcox et al., 2014), confirming its key role in self‐regulating alcohol consumption. Measures that are directly sensitive to neural activity can provide insight into the neural underpinnings of cognitive control, as reflected in cognitive conflict and response execution.

Event‐related theta power (erTP, 4–7 Hz) has emerged as a key neural index of the engagement of cognitive control (Cavanagh & Frank, 2014; Hanslmayr et al., 2008; Marinkovic et al., 2019; Rosen et al., 2016). Prior research has shown that acute alcohol intoxication reduces erTP in high‐conflict situations (Beaton et al., 2018; Kovacevic et al., 2012; Marinkovic et al., 2019; Rosen et al., 2016), making it a valuable marker for detecting potential impairments of decision making. While studies on people with AUD have also reported reduced erTP, indicating compromised engagement of cognitive and inhibitory control processes (Harper et al., 2018a; Meyers et al., 2023; Pandey et al., 2012), the evidence in BDs is scant and limited to target detection, response inhibition, and memory tasks (Correas et al., 2019; Holcomb et al., 2019; Huang et al., 2022). Thus, the impact of binge drinking on theta power during decision making remains largely unexplored. To advance our understanding of the neural underpinnings of these processes, we have analyzed erTP as a marker of cognitive control engagement during a modified Stroop word‐color naming task.

Event‐related beta power (erBP, 15–25 Hz) changes are most prominent over the sensorimotor cortices and reflect the engagement of the network involved in response preparation, inhibition, and execution (Barone & Rossiter, 2021). erBP decreases, or desynchronizes, in anticipation and preparation for movement and increases during motor inhibition and is associated with impulsivity (Barone & Rossiter, 2021; Barth et al., 2021; Happer et al., 2024; Tzagarakis et al., 2019). However, research on the impact of alcohol on beta activity is lacking. Beaton et al. (2018) reported a notable decrease in theta power during acute alcohol intoxication as a function of cognitive control demands, but only subtle effects on beta desynchronization. Importantly, no interactions between beverage and task conditions were observed, indicating that beta activity was not differentially engaged by cognitive conflict. Similarly, young BDs showed lower erTP on inhibitory NoGo trials, which correlated with alcohol intake. In contrast, an overall early decrease of erBP was indicative of a deficient “inhibitory brake” before deliberate motor response (Holcomb et al., 2019). These findings imply that alcohol interferes primarily with cognitive processing, while additionally affecting motor preparation, inhibition, and execution more broadly. To address the lack of evidence in BDs during cognitive control tasks, we investigate the impact of binge drinking on engagement of motor‐related processes during response conflict.

Despite known sex differences in alcohol metabolism, faster progression of AUD, and greater alcohol‐related harms observed in women than in men, research on the neurobehavioral indices of cognitive control as a function of alcohol misuse and biological sex is limited (Nixon et al., 2014; Ruiz & Oscar‐Berman, 2013). In studies of inhibitory control, lower erTP was associated with drinking in young women, but not in men (Harper et al., 2018b), consistent with other reports of women's elevated vulnerability to the adverse effects of alcohol on cognitive functions (Nixon et al., 2014; Ruiz & Oscar‐Berman, 2013). Conversely, impulsivity, known to play a key role in the development and persistence of alcohol misuse, may differentially influence alcohol consumption and the associated neurocognitive outcomes in men vs. women (Mitchell & Potenza, 2015; Weafer, 2020). These potentially sex‐dependent effects could manifest as sex‐specific neural responses during decision making, response preparation, and execution, as a function of cognitive control demands and BD history. Although the extant evidence is rudimentary, it highlights the importance of considering sex differences in the studies of cognitive control in young adult BDs.

The overall aim of the current study was to explore the impact of binge drinking on cognitive control as reflected in task performance and event‐related oscillatory activity during a Stroop naming task in young adults. The biological sex factor was also modeled in the statistical analyses. Scalp EEG signals were analyzed as theta and beta event‐related oscillations, with a focus on their roles in cognitive and motor control processes, respectively. The neural indices were analyzed in conjunction with task performance and an array of alcohol‐related variables and measures of anxiety, depression, personality, and cognitive functions.

## MATERIALS AND METHODS

### Participants

Sixty‐eight participants (23.37 ± 3.23 years of age, 34 women) were recruited from San Diego State University and the surrounding community with flyers and advertisements. Interested individuals filled out an online screening questionnaire that assessed their handedness, alcohol and substance use habits, neurological or neuropsychiatric disorders, and perceptual or learning impairments. The survey was followed by an interview to confirm eligibility for the study. All participants who met the inclusionary criteria were right‐handed adults who reported no tobacco, cannabis, or illicit drug use within the past month, no history of brain injury, seizures, neurological or neuropsychiatric disorders, no vision, hearing, or learning problems, and no medication use at the time of the study. An overview of the sample characteristics is presented in Table 1. Participants who reported five or more binge episodes within the past 6 months were classified as BD (*N* = 34), whereas LD (*N* = 34) reported no more than one binge episode in that period. A binge episode was defined as consuming 6+ (men) or 5+ alcoholic drinks (women) within 2 h. This criterion was based on the evidence indicating that the legal BAC limit is more likely to be reached at that level of consumption (Lange & Voas, 2001). Abstainers were not recruited into the study, and all participants reported drinking at least one drink per week on average in the past 6 months. The two groups were matched on age, biological sex, ethnicity, and educational background. The study's protocol was approved by the Human Research Protection Programs at the University of California, San Diego, and San Diego State University. All participants provided written informed consent to participate in the study and received monetary compensation for their participation.

**TABLE 1:** Participant characteristics for Binge Drinking (BD) and Light Drinking (LD) groups.

	BD (*n* = 34)	LD (*n* = 34)	U/X^2^	*p*
Women %	50%	50%	0.015^A^	0.901
Age (years)	23.32 ± 3.27	23.41 ± 3.23	607	0.716
% White/Non‐Hisp	68%	74%	0.350^A^	0.839
Family Hist. of AUD (%)	59%	41%	1.812^A^	0.404
Education years	15.67 ± 2.00	16.29 ± 2.28	701	0.128
Undergraduate GPA	3.15 ± 0.46	3.46 ± 0.37	346	**<0.05**
BMI	24.92 ± 3.89	23.25 ± 3.14	450	0.115
In the past 6 months				
Drinking days/week	3.13 ± 1.20	1.65 ± 0.91	124	**<0.001**
Drinks/occasion	5.56 ± 1.74	2.09 ± 1.10	23	**<0.001**
Drinks/week	17.93 ± 10.70	3.70 ± 2.82	16	**<0.001**
Max no. of drinks in 24 h	12.73 ± 5.43	4.79 ± 1.92	26	**<0.001**
Binge episodes	14.75 ± 13.17	0.09 ± 0.28	0	**<0.001**
Alcohol‐related blackouts	4.40 ± 3.43	0.03 ± 0.17	3	**<0.001**
Age of drinking onset	16.00 ± 1.44	18.55 ± 2.02	184	**<0.001**
Drinks to feel effects (SRE)	5.54 ± 1.36	4.31 ± 1.34	277	**<0.001**
Drinking motives (DMQR‐R SF)				
Enhancement	6.64 ± 1.34	5.32 ± 1.49	305	**0.001**
Social	7.55 ± 1.30	6.24 ± 1.61	308	**0.001**
Conformity	4.24 ± 1.41	4.06 ± 1.21	529	0.663
Coping	4.97 ± 1.71	3.76 ± 0.96	308	**<0.001**
Drinking conseq. (B‐YAACQ)	10.58 ± 5.20	1.91 ± 1.87	62	**<0.001**
Severity of alcoholism (SMAST)	3.52 ± 3.54	0.53 ± 0.85	257	**<0.001**
Anxiety (GAD‐7)	4.03 ± 5.04	2.44 ± 3.08	465	0.217
Depression (PHQ‐9)	4.39 ± 4.72	2.09 ± 1.98	430	0.093
Personality (EPQ)				
Neuroticism	3.76 ± 3.28	3.50 ± 3.22	524	0.640
Psychoticism	2.55 ± 2.06	2.18 ± 1.62	512	0.527
Extraversion	9.63 ± 2.28	8.29 ± 3.37	450	0.157
Impulsivity (ABIS)	2.08 ± 0.45	1.84 ± 0.31	385	**<0.05**
NIH cognitive battery				
Working memory	0.76 ± 0.10	0.74 ± 0.11	401	0.353
Dimensional shift	0.87 ± 0.14	0.92 ± 0.05	579	0.097
Processing speed	0.58 ± 0.07	0.60 ± 0.09	546	0.236
Episodic memory	0.76 ± 0.19	0.80 ± 0.15	504	0.416

*Note*: *M* ± SD were calculated for all measurements. Group comparisons for categorical variables (marked with A) were tested with the chi‐squared (*X* ^2^) test and all others were analyzed with the nonparametric Mann–Whitney U‐test. Significant *p*‐values are reported in boldface.

Abbreviations: ABIS, Abbreviated Impulsiveness Scale; BMI, body mass index; BSSS, Brief Sensation Seeking Scale; DMQ‐R, Drinking Motive Questionnaire Revised; EPQ, Eysenck Personality Questionnaire; GAD‐7, Generalized Anxiety Disorder scale; GPA, grade point average; PHQ‐9, Patient Health Questionnaire assesses depression severity; SMAST, Short Michigan Alcoholism Screening Test; SRE, self‐report of the effects of alcohol.

### Procedure

Upon arrival, participants completed a timeline follow‐back (TLFB; Sobell & Sobell, 1992) to establish their recent alcohol consumption patterns within the past month and to confirm abstaining from drinking for at least 2 days before the study. They completed a questionnaire battery that assessed their medical history and alcohol‐related behaviors within the past 6 months, age at drinking onset, anxiety (GAD‐7; Spitzer et al., 2006), depression (PHQ‐9; Kroenke & Spitzer, 2002), personality (EPQ; Eysenck et al., 1985), and impulsivity (ABIS; Coutlee et al., 2014). Participants also completed the Self‐Report of the Effects of Alcohol (SRE; Schuckit et al., 1997), the Drinking Motive Questionnaire Revised (DMQ‐R SF; Kuntsche & Kuntsche, 2009), the Short Michigan Alcoholism Screening Test (SMAST; Selzer et al., 1975), and the NIH‐Toolbox Cognitive Battery (Weintraub et al., 2013), which assesses working memory capacity (List Sorting Working Memory Test), cognitive flexibility (Dimensional Change Card Sort, DCCS), processing speed (Pattern Comparison Processing Speed Test), and episodic memory (Picture Sequence Memory, PSM). Participants were screened for drug use using a 12‐panel urine multidrug test (Discover, American Screening Corporation), and all results were negative.

### Experimental paradigm

Cognitive control was probed with a modified version of the Stroop task that combines color naming and reading (Kovacevic et al., 2012; Marinkovic et al., 2019; Molnar et al., 2018). Four color words (*red, green, blue, and yellow*) were presented individually on a computer screen. Participants were instructed to press one of four buttons corresponding to the color of the font of each word written in color. In the congruent condition (CONG, 25% of trials), the word meaning matched the font color (e.g., *red* written in red), eliciting low response conflict. In the incongruent condition (INCONG, 25% of trials), the word meaning did not match the font color (e.g., *red* written in yellow). The mismatch elicited high response conflict and engaged cognitive control to override the automatic reading response. In the third condition (READ, 50% of trials), color words were written in gray font, and participants were asked to press a button corresponding to the *meaning* of the word. This control condition elicited a low level of response conflict and helped maintain reading dominance and automaticity. Participants responded with both hands on a four‐button device (Current Designs, Inc.), which contained red, green, blue, and yellow buttons, with finger‐response mapping from left to right as follows: the left middle and index fingers pressed the red and green buttons, respectively, and the right index and middle fingers pressed the blue and yellow buttons, respectively.

Using the Presentation software package (Version 18.1; www.neurobs.com), a total of 480 trials (120 CONG, 120 INCONG, 240 READ) were presented in a randomized order on a black background at a viewing distance of 180 cm from a 24‐inch color monitor with a visual angle of 3.02° by 1.25°. All colors were equally represented across all conditions, and participants pressed buttons with all fingers equally often. Each word was presented for 300 ms in capital letters, followed by a fixation string (+++++) in gray, which remained on the screen until the next trial. The stimulus onset asynchrony (SOA) was 2200 ms with a ± 200 ms jitter in increments of 50 ms to mitigate trial onset predictability. Before the experiment, participants were thoroughly familiarized with the task by completing a 3‐step practice session.

### EEG signal acquisition and analysis

The EEG signal was sampled continuously at 500 Hz with a 64‐channel Brain Vision system (Brain Products GmbH, Germany) with impedance below 5 kΩ. Electrodes placed on the nose and forehead served as the reference and ground, respectively. A bipolarly referred vertical electrooculogram (EOG) was recorded to monitor eye blinks.

Continuous data were bandpass‐filtered from 0.1 to 100 Hz and segmented into epochs extending from −300 to 1000 ms relative to stimulus onset. A 300 ms padding interval was added to the beginning and end of each epoch to account for edge artifacts resulting from the Morlet wavelet convolution (Kovacevic et al., 2012; Marinkovic, Rosen, et al., 2012; Oostenveld et al., 2011). Data were analyzed using customized MATLAB (MathWorks, Natick, MA) routines, which incorporate publicly available Fieldtrip (Oostenveld et al., 2011) and EEGLAB toolboxes (Delorme & Makeig, 2004). Trials with evident artifacts were removed by careful visual inspection and threshold‐based rejection. An independent component analysis (Delorme & Makeig, 2004) was used to identify and remove artifacts caused by eye blinks and heartbeats. Only correct responses were included in the analysis.

EEG data were analyzed in the time‐frequency domain by calculating complex power spectra using Morlet wavelets within the theta (4–7 Hz) and beta (15–25 Hz) frequency ranges (Beaton et al., 2018; Holcomb et al., 2019; Kovacevic et al., 2012; Marinkovic et al., 2019). The wavelet results were additionally inspected for artifacts, and the padding was removed. erTP and erBP were calculated as the percent signal change from the prestimulus baseline (−300 to 0 ms) for each condition. Given that the Stroop task is language‐based, we examined the expected left‐dominance (Marinkovic, Rickenbacher, et al., 2012) by analyzing erTP within the left central (FC5, FC3, FC1, C5, C3, C1, CP5, CP3, and CP1) and right central (FC6, FC4, FC2, C6, C4, C2, CP6, CP4, and CP2) electrode clusters that were averaged into regions of interest. erBP was analyzed by averaging the signals from electrodes over the sensorimotor region (C2, C3, C4, and C5) (Beaton et al., 2018). erTP subtractions were calculated for the Stroop effect to isolate the effects evoked by high vs. low levels of conflict interference. Specifically, the two types of Stroop effect contrasts were defined as Incongruent minus Congruent (I‐C) and Incongruent minus Read (I‐R) differences. R Studio version 4.4.0 was used for statistical analyses.

## RESULTS

### Participant characteristics

As shown in Table 1, BDs and LDs were matched on age, biological sex, ethnicity, family history of AUD, and education. The two groups did not differ in personality, anxiety, or cognitive functions, including processing speed, working memory, and episodic memory. BDs tended to report more symptoms of depression and had marginally weaker cognitive flexibility, as assessed with the Dimensional Change Card Sort test as part of the NIH‐Toolbox Cognitive Battery. They had a lower GPA and tended to weigh more than their LD counterparts. As expected, BDs exceeded LDs on all drinking‐related measures, reporting higher levels of habitual and high‐intensity consumption. They started drinking at an earlier age and showed greater impulsivity.

### Task performance

#### Accuracy

**FIGURE 1:**
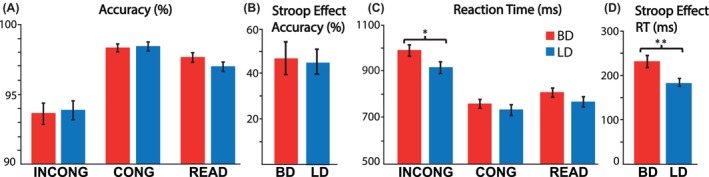
Performance measures (means ± standard errors) for: (A) Task accuracy, (B) Stroop effect for accuracy (CONG‐INCONG), (C) reaction times, and (D) Stroop effect for reaction times (INCONG‐CONG) for binge (BDs) and light drinkers (LDs). BDs took longer to respond to the high‐conflict incongruent condition but performed as accurately as LDs. *p < 0.05; **p < 0.01).

Behavioral responses were analyzed using a mixed ANOVA model: 2 (group; BD vs. LD) × 2 (biological sex; men vs. women) × 3 (condition; CONG vs. INCONG vs. READ). This task version was effective in eliciting word‐color interference overall, as reflected in lower accuracy in the INCONG condition than in the CONG (*t*_64_ = 4.64; *p* < 0.001) and READ conditions (*t*_64_ = 3.57; *p* < 0.001). However, performance accuracy was equivalent between BDs and LDs across all conditions (*F*_1,64_ = 0.04; *p* = 0.83; Figure 1A). Similarly, the two groups did not differ on the Stroop effect (Figure 1B; CONG ‐ INCONG subtraction) (*t*_64_ = 0.14, *p* = 0.89). The number of drinks consumed per occasion correlated negatively with average accuracy for BDs (*r* = −0.35, *p* = 0.04) but not for LDs (*r* = −0.18, *p* = 0.32). Similarly, the number of drinks per occasion correlated positively with the accuracy of the Stroop effect in BDs (*r* = 0.53, *p* = 0.001) but not LDs (*r* = 0.22, *p* = 0.22). These correlations suggest that only for BDs, heavier drinking was associated with poorer overall performance and a larger Stroop interference effect, indicating that greater alcohol consumption might be linked to increased difficulty resolving cognitive conflict on high‐interference trials.

#### Reaction times

RTs additionally confirmed that the Stroop task was effective in inducing cognitive interference. A main effect of condition (*F*_2,128_ = 441.78, *p* < 0.001) revealed longer RTs in the INCONG than in the CONG (*t*_64_ = 206.70, *p* < 0.001) and READ conditions (*t*_64_ = 165.10, *p* < 0.001) overall (Figure 1C). Furthermore, an interaction between group and condition (*F*_2,128_ = 5.67, *p* = 0.004) indicated that BDs exhibited longer RT only in the INCONG condition (*t*_66_ = 2.13, *p* = 0.04) than LDs. This was also evident in the Stroop effect (Figure 1D, INCONG–CONG subtraction), which demonstrated that BDs were more susceptible to cognitive interference than LDs (*t*_64_ = 3.20, *p* = 0.002). Longer RTs only on INCONG trials in the BD group are suggestive of the speed‐accuracy trade‐off to maintain normative performance accuracy under challenging conditions. High‐intensity drinking, quantified as the maximum number of drinks within 24 h in the past 6 months, correlated with average RT (*r* = 0.26, *p* = 0.04) as well as the RT for the Stroop effect (*r* = 0.39, *p* = 0.001). These results suggest that participants with higher levels of alcohol intake took longer to respond and experienced greater cognitive interference. No sex differences were found for performance accuracy or RTs.

### Event‐related theta power (erTP)

**FIGURE 2:**
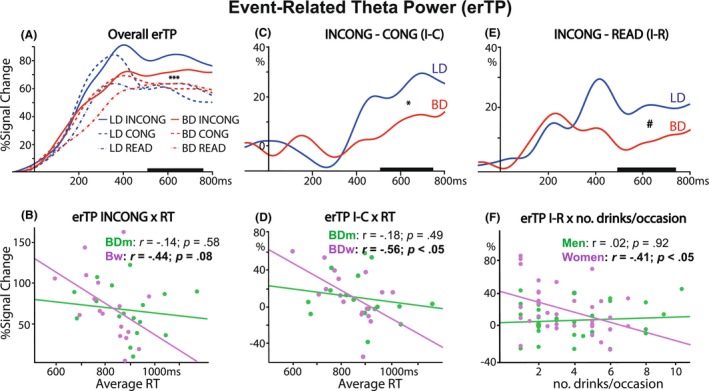
Event‐related theta power (erTP) over the left electrode cluster showing the effects of group and condition (top panel), and associations with performance and alcohol consumption for men and women (bottom panel). (A) Across all participants, theta power was greater in the INCONG condition than in the CONG and READ conditions, reflecting increased cognitive control demands. (B) On high‐conflict (INCONG) trials, erTP was negatively correlated with response times (RT) among binge drinker (BD) women, but not among BD men. (C) A main effect of group was observed for the erTP Stroop effect (I‐C) contrast, with BDs exhibiting lower theta power than light drinkers (LDs). (D) Lower theta power in the I‐C contrast was associated with slower RTs only among BD women. (E) BDs exhibited marginally lower theta power than LDs for the erTP I‐R contrast, (F) which was negatively associated with levels of habitual drinking for women only. erTP is expressed as a percent change in power from the prestimulus baseline. It was analyzed in the 500–750 ms time window, as marked on the *x*‐axis. *p < 0.05; ***p < 0.001; ^#^ *p* = 0.054.

Engagement of cognitive control as a function of binge drinking history and sex was evaluated with erTP. As shown in Figure 2, erTP increased after stimulus onset during sensory and early lexico‐semantic processing, followed by engagement of cognitive control at a later stage. To capture erTP during response conflict, we analyzed a 500–750 ms time window, in line with previous studies using similar paradigms (Kovacevic et al., 2012; Rosen et al., 2016). One participant was excluded from the erTP analysis because their data deviated significantly from the rest of the data set. A mixed ANOVA model: 2 (group) × 2 (sex) × 3 (condition) × 2 (hemisphere) revealed a main effect of hemisphere. Task‐related theta power was greater over the left than the right hemisphere cluster (*F*_1,62_ = 5.18; *p* = 0.03), indicating sensitivity to the effects of group, sex, and conditions. Therefore, the results presented here and in Figure 2 refer to erTP over the left hemisphere. As shown in Figure 2A, a main effect of condition (*F*_2,126_ = 17.75, *p* < 0.001) was observed, with higher erTP in the INCONG than in the CONG (*t*_63_ = 0.18, *p* < 0.001) and READ (*t*_63_ = 0.14, *p* < 0.001) conditions across all participants. For the overall erTP, there was no main effect of group (*F*_1,63_ = 0.06; *p* = 0.81). However, a marginal group‐condition interaction (*F*_2,126_ = 2.90; *p* = 0.06) revealed conflict‐related effects only in LDs. More specifically, erTP was higher in the INCONG condition than in the CONG (*t*_63_ = 0.26, *p* < 0.001) and READ (*t*_63_ = 0.20, *p* < 0.001) conditions in the LD group. Higher theta correlated with faster RTs (*r* = −0.31, *p* = 0.01), confirming that erTP is an index of more effective engagement of cognitive control. This association was marginally significant only for BD women (*r* = −0.44, *p* = 0.08) but not for BD men (*r* = −0.14, *p* = 0.58) (Figure 2B).

The effects attributable to conflict interference were tested for the Stroop effect, as reflected in I‐C and I‐R contrast conditions, which were derived by subtracting low‐conflict from high‐conflict trials. The main effect of group for the I‐C condition (*F*_1,63_ = 4.10; *p* = 0.047) revealed lower conflict‐specific theta power in BDs than in LDs (Figure 2C). Among BD women, lower I‐C theta power was significantly associated with slower RT (*r* = −0.56, *p* = 0.02), an effect not observed in BD men (*r* = −0.18, *p* = 0.49; Figure 2D). The main effect of group was also evident in the I‐R condition (Figure 2E; *F*_1,63_ = 3.84; *p* = 0.05). Additionally, a main effect of sex (*F*_1,63_ = 5.25; *p* = 0.03) and a group‐sex interaction (*F*_1,63_ = 4.24; *p* = 0.04) were found for the I‐R condition. Post hoc tests revealed that erTP was lower only for BDw than for LDw (*t*_63_ = 0.24; *p* = 0.03), while it was higher for LDw than for LDm (*t*_63_ = 0.26; *p* = 0.02). Theta power in the I‐R condition was negatively associated with number of drinks per occasion among women (*r* = −0.41, *p* = 0.02), but not men (*r* = 0.02, *p* = 0.92), suggesting that greater habitual alcohol use was linked to reduced neural engagement during conflict processing for women only (Figure 2F).

**FIGURE 3:**
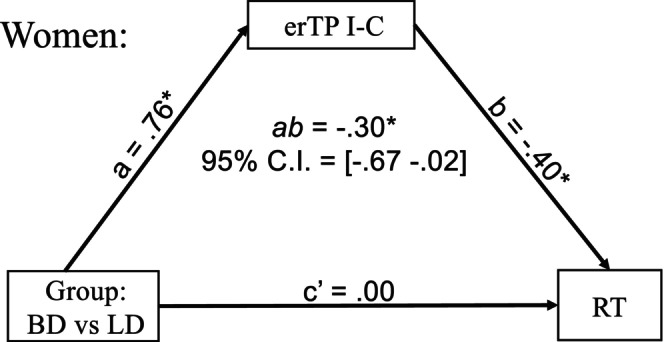
Mediation analysis examined sex‐specific associations between the Stroop effect (INCONG–CONG) event‐related theta power (erTP) and reaction times. For women, group (binge drinker [BD] vs. light drinker [LD]) differences in response times (RTs) are completely mediated by I‐C erTP, with no direct group effect on RT. This finding suggests that weaker conflict‐related engagement of cognitive control in BD women results in degraded behavioral task performance. * indicates a statistically significant effect (95% CI excludes zero). All parameters for both sexes can be found in the Appendix S1.

To further explore the observed sex‐specific associations between theta power and behavioral outcomes, we used a multiple‐group mediation model for women and men separately (Ryu, 2015). We tested whether the erTP to the I‐C Stroop effect mediated the impact of group (BD vs. LD) on response times (RT). Model fit was excellent (CFI = 1.000, RMSEA < 0.001, SRMR < 0.001). As shown in Figure 3, the confidence interval for the indirect mediation effect does not include zero, indicating significant mediation by erTP among women but not among men. The observed sex‐specific pathway linking binge drinking to RT impairments via erTP indicates that weaker conflict‐related engagement of cognitive control in BD women results in performance deficits. All parameters of the multiple‐group mediation results for men and women are presented in Appendix S1.

### Event‐related Beta power (erBP)

**FIGURE 4:**
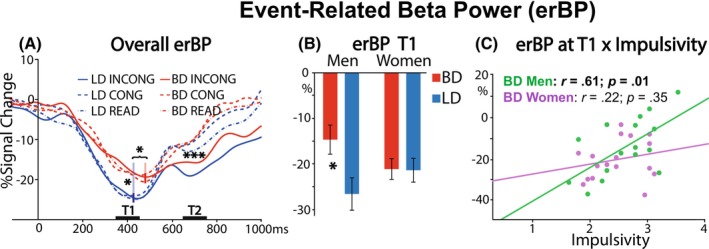
Event‐related beta power (erBP) as a function of group, biological sex, and Stroop conditions. erBP was calculated as the average across electrodes C2, C3, C4, and C5. The stage of sensorimotor system engagement was examined in the 350 to 450 ms time window (T1), as marked on the *x*‐axis. (A) erBP decrease (desynchronization) was less pronounced and delayed in binge drinkers (BDs) compared with light drinkers (LDs) in T1. Conflict‐specific response preparation was captured in a 650–750 ms time window (T2). In T2, the erBP decrease was greater in the INCONG than in both low‐conflict conditions overall. Vertical lines represent erBP nadir points for BD and LD groups, indicating a delayed engagement of the sensorimotor system in BDs. (B) In T1, erBP desynchronization was lower in BD men than in LD men, while erBP did not differ for women. erBP is shown as a percent change in power from the baseline within the 15–25 Hz band. *p < 0.05; ***p < 0.001. (C) erBP in T1 correlated with impulsivity only among BD men.

To examine the impact of group, sex, and response conflict on the stages of sensorimotor engagement, we analyzed erBP (averaged across electrodes C2, C3, C4, and C5) with a 2 × 2 × 3 mixed ANOVA. A T1 time window (350–450 ms latency) captured the nadir of beta power, reflecting early response preparation (Figure 4). In T1, BDs showed less beta decrease than LDs (*F*_1,64_ = 4.03, *p* = 0.049), with a marginal group‐sex interaction (*F*_1,64_ = 3.68, *p* = 0.06). Post hoc tests indicated that erBP differed only between BD and LD men (*t*_64_ = 0.12; *p* = 0.04) (Figure 4B). Furthermore, for BD men, erBP correlated with impulsivity as measured by ABIS (*r* = 0.61, *p* = 0.01), indicating that a lower decrease in erBP was associated with greater impulsivity, as shown in Figure 4C. BD women did not display this association (*r* = 0.22, *p* = 0.35). Importantly, impulsivity scores did not differ between men and women, even though impulsivity was higher in BDs than in LDs (Table 1). The association between lower erBP decrease and impulsivity remained significant for the BD group overall (*r* = 0.48, *p* = 0.01), but no such correlation was found for the LD group (*r* = −0.04, *p* = 0.81). In contrast, the EPQ Psychoticism trait, which is associated with impulsivity, was negatively correlated with erBP for LDs (*r* = −0.42, *p* = 0.01), but not for BDs (*r* = 0.07, *p* = 0.69). Importantly, no effects of condition were observed, confirming that erBP in T1 is sensitive to motor response readiness and not to cognitive processing.

**FIGURE 5:**
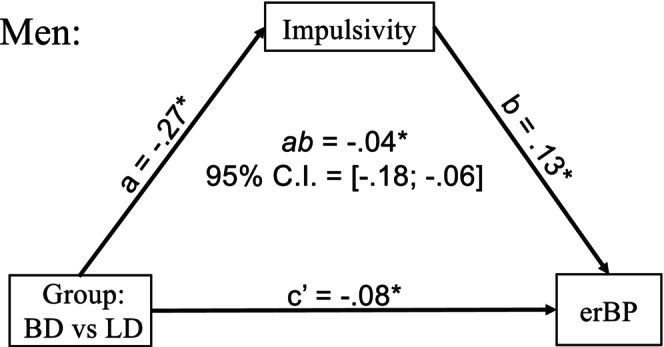
Mediation analysis shows that, for men only, impulsivity partially mediates group (binge drinker [BD] vs. light drinker [LD]) differences in event‐related beta power (erBP), in addition to a direct impact of group on erBP. * indicates a statistically significant effect (95% CI excludes zero). All parameters for both sexes can be found in the Appendix S1.

We conducted a multiple‐group mediation analysis (Ryu, 2015) to explore further the mechanisms underlying the impact of binge drinking on associations between impulsivity and erBP in T1 for men and women (Figure 5). Model fit was excellent (CFI = 1.000, RMSEA < 0.001, SRMR < 0.001). The analysis revealed sex‐specific mediation patterns, such that impulsivity mediated group differences in beta power only for men. However, this mediation effect only partially accounted for group differences in erBP since a direct effect of group (c′) was also significant. These results suggest that while impulsivity contributes to the group differences in beta desynchronization among men, a history of binge drinking exerts an independent effect on erBP. A table with all parameters of the multiple‐group mediation model for both sexes is included in Appendix S1.

In BDs, the erBP decrease was lower and unfolded more slowly. An ANOVA of the nadir latencies within T1 revealed a slower erBP desynchronization in BDs than in LDs (*F*_1,64_ = 5.26, *p* = 0.03), Figure 4A. The latency of the first nadir correlated with I‐C RT (*r* = 0.26, *p* = 0.03). Furthermore, beta nadir latency correlated with the number of binge episodes for men (*r* = 0.35, *p* = 0.04), but not for women (*r* = 0.26, *p* = 0.14), suggesting that high‐intensity drinking dysregulates and delays response preparation in men.

The T2 time window (650–750 ms) was sensitive to conflict‐specific engagement of the motor circuitry. As shown in Figure 4A, a main effect of condition (*F*_2,128_ = 20.47, *p* < 0.001) was due to a greater erBP decrease for the INCONG than the CONG (*t*_64_ = 0.01; *p* < 0.001) and READ conditions (*t*_64_ = 0.01; *p* < 0.001). This is consistent with prolonged engagement of the motor control network and longer RTs on the INCONG trials. Indeed, the T2 nadir latency correlated positively with the RT Stroop interference effect (*r* = 0.32, *p* = 0.01) overall.

## DISCUSSION

This study examined neurobehavioral characteristics of cognitive control as a function of binge drinking in young adult men and women. A modified Stroop task probed conflict‐related decision making through behavioral and neural metrics. Theta and beta oscillatory activity revealed how the cognitive and motor response aspects of cognitive control are implemented, respectively, in real time. Given the vulnerability of the cognitive control circuits to hazardous drinking and addiction, we examined associations of neurobehavioral indices with drinking behaviors and other self‐reported variables. The principal results can be summarized as follows: (1) Task accuracy was equivalent between the two groups, but BDs took longer to respond on high‐conflict incongruent trials (Figure 1), which was associated with heavier drinking. (2) Theta activity reliably indexed cognitive control engagement, and conflict‐specific erTP was reduced in BDs relative to LDs (Figure 2). (3) Theta alterations were sex‐dependent: reduced conflict‐related erTP in BD women was associated with alcohol use and response slowing and accounted for group performance differences (Figures 2 and 3). (4) Beta power during response preparation showed group differences, with BDs exhibiting smaller and slower erBP decreases than LDs (Figure 4). (5) Beta power effects were sex‐dependent: erBP alterations and their mediation by impulsivity were present only in men but not women (Figures 4 and 5). Overall, these findings indicate dysregulated cognitive control, especially in BD women, reflecting suboptimal neural integration of conflict‐relevant representations and an inability to optimize decisions in challenging situations. In contrast, impulsivity mediates engagement of the motor response system, especially in BD men. The link between impulsivity and dysregulated motor readiness in BD men may be relevant to their inability to refrain from risky behaviors. The results emphasize the need to consider biological sex as a moderating factor in the neural mechanisms of cognitive control in BDs.

Measures of task performance indicated that BDs were especially susceptible to Stroop interference, requiring more time to achieve the same level of accuracy as LDs on high‐conflict trials (Figure 1). Indicative of executive deficits, this finding is consistent with similar reports in BDs (Molnar et al., 2018) and individuals with AUD (Fadardi & Cox, 2006). In contrast to rather subtle task performance deficits in BDs, EEG indices detected neural alterations associated with alcohol misuse more readily, given their high sensitivity to neural activity. Overall erTP increased as a function of conflict across conditions (Figure 2A), but group differences emerged only at the level of conflict‐specific contrasts, with BDs showing reduced erTP relative to LDs (Figure 2C,E). This finding suggests that the recruitment of cognitive control to override automatic word reading in favor of controlled processing is impaired in BDs. This is in line with reports of lower conflict‐related theta power during acute intoxication than placebo (Kovacevic et al., 2012; Marinkovic et al., 2019; Rosen et al., 2016), suggesting that the detrimental effects of inebriation on executive functions extend beyond the intoxication episode. Notably, individuals with AUD also exhibit reduced erTP during conflict‐inducing tasks (Harper et al., 2018a; Meyers et al., 2023; Pandey et al., 2012). The confluence of behavioral and neural indices of deficient cognitive control in young adult BDs who misuse alcohol at subclinical levels converges with other similar reports of reduced erTP in BDs during cognitive tasks (Correas et al., 2019; Holcomb et al., 2019; Huang et al., 2022). Given that erTP can be viewed as an overarching oscillatory index of cognitive engagement and integration, lower erTP suggests that binge drinking is associated with diminished capacity to make decisions under conflict. In contrast, fMRI studies probing cognitive control have shown *greater* BOLD activity in BDs and in abstinent individuals with AUD (Alderson Myers et al., 2021; Molnar et al., 2018; Wilcox et al., 2014). These seemingly diametrically opposite findings may reflect fundamentally different biophysical properties of the EEG and BOLD signals. They also suggest that elevated BOLD activity may reflect compensatory engagement, serving to maintain normative functioning despite neural degradation. Furthermore, it is consistent with allostatic changes in the neural excitation/inhibition balance due to alcohol misuse (Koob, 2013; Koob & Le Moal, 2008).

Importantly, lower erTP to cognitive conflict was especially evident in BD women, resulting in group differences between BD and LD. Only in women, conflict‐related erTP mediated the link between binge drinking and slower RT (Figure 3). This finding suggests that reduced cognitive control in BD women may impair their decision‐making capacity. However, given the small sample size, these findings merit replication and should be interpreted with caution. Nonetheless, the current study supports other evidence suggesting that, despite their lower alcohol intake, women are more vulnerable to the detrimental impact of binge drinking on neurocognitive functions (Harper et al., 2018b; Nixon et al., 2014; Ruiz & Oscar‐Berman, 2013). This is compounded by recent trends suggesting a narrowing of sex differences in alcohol intake, as women's rates of consumption, binge drinking, and AUD approach those of men, especially among young adults (White, 2020).

As an executive function, cognitive control is primarily engaged by ambiguous situational demands that require making deliberate decisions in the service of flexible behavior optimization. As described above, task‐related theta is sensitive to the detection of incongruity and to top–down regulatory control over relevant action choices (Cavanagh & Frank, 2014; Marinkovic et al., 2019; Rosen et al., 2016). At the same time, anticipatory preparation for movement proceeds in parallel in an interactive manner. Generated by the motor system, preparatory activity is automatic and largely inaccessible to conscious awareness (Beaton et al., 2018). It is reflected in an event‐related decrease in beta power, which indicates anticipatory response readiness (Barone & Rossiter, 2021; Beaton et al., 2018; Happer et al., 2024). This pattern was confirmed in the present study, as stimuli induced an expected early overall erBP decrease reflecting involuntary motor preparation (T1 in Figure 4A). The erBP decrease was attenuated and delayed in BDs compared with LDs. Reduced beta desynchronization is consistent with findings in intoxicated social drinkers during a similar task that evoked response conflict (Beaton et al., 2018). Clinical studies have linked reduced beta decrease to motor system impairments and executive deficits in various disorders affecting motor function, as well as in healthy and pathological aging (Peter et al., 2022). Reduced erBP desynchronization is commonly interpreted as a marker of dysregulated engagement of the neural circuitry necessary for adaptive motor inhibition and execution. This broad conclusion may also apply to BDs in this study, which provides novel mechanistic evidence on the impact of repeated binge drinking on motor engagement and the underlying alterations in cell signaling.

Notably, a marginal group‐sex interaction in the early (T1) time window revealed that these effects primarily pertained to BD men (Figure 4B), who exhibited a reduced erBP decrease, which was associated with greater impulsivity. Among men, group differences in erBP were partially mediated by impulsivity, a trait consistently linked to greater alcohol use and increased risk for AUD (Mitchell & Potenza, 2015). In addition, a remaining direct group effect indicated that engaging in binge drinking episodes is likely to disrupt neural mechanisms involved in motor engagement beyond the influence of impulsivity. These findings point to erBP as a promising neurophysiological marker for understanding cognitive‐motor impairments associated with binge drinking. Crucially, the fact that these effects were observed predominantly in men, despite the absence of sex differences in impulsivity, indicates that men might be especially at risk for developing motor control deficits as a result of repeated binge drinking.

In the current study, BDs reported higher impulsivity than LDs, which appears to contradict studies showing greater beta desynchronization in more impulsive individuals (Barth et al., 2021; Happer et al., 2024; Tzagarakis et al., 2019). Two points help reconcile this seeming contradiction. First, in the LD group of this study, we observed the expected pattern: greater erBP decrease was associated with higher scores on the Psychoticism (P) scale of the EPQ questionnaire, which has been linked to trait impulsivity (Rawlings, 1984). The opposite relationship emerged in the BD group, in which higher impulsivity was associated with less beta desynchronization. This suggests that binge drinking alters the typical association between impulsivity and greater erBP decrease. Second, the task demands differ: previous studies used paradigms requiring fast responses in the context of motor inhibition, whereas our Stroop task emphasizes cognitive control and interference resolution.

### Limitations

Overall, this study provides novel insights into sex‐based differences in neural mechanisms underlying cognitive control deficits in young adult BDs. However, findings from the current study should be considered in light of potential limitations that should be addressed in future research. Given the relatively low statistical power of our sex‐stratified analyses, these findings should be interpreted with caution. Future studies with larger samples are needed to confirm the robustness of these sex‐specific effects. Furthermore, given its cross‐sectional design, this study cannot address whether the observed effects reflect premorbid traits or result from heavy drinking. A prospective, longitudinal approach is needed to address such concerns (https://abcdstudy.org/). However, alcohol‐related variables correlated with neural alterations in BDs, suggesting their vulnerability to neurotoxicity. Additionally, this study did not include an objective verification of alcohol abstinence at the time of testing. While participants reported no alcohol use for at least 2 days before the experimental session, future studies would also benefit from including a breathalyzer screening to ensure full compliance with abstinence instructions. Relatedly, participants' AUD and substance use disorder status need to be evaluated with standardized diagnostic assessments to characterize their addiction profiles better and permit comparisons with clinical samples.

## CONCLUSION

Taken together, this study demonstrates that binge drinking disrupts core components of decision making through cognitive and motor control pathways. Although BDs matched LDs in performance accuracy, their longer RT on high‐conflict trials indicate compensatory slowing under increased demands on cognitive control. EEG analysis revealed reduced conflict‐specific erTP in BDs compared with LDs, indicative of weaker cognitive control engagement, especially in BD women. Lower conflict‐induced erTP correlated with alcohol consumption and mediated the impact of binge drinking on degraded task performance in BD women. Given its role as an index of cognitive engagement and integration, reduced erTP in BDw is suggestive of their impaired capacity to make decisions in cognitively challenging situations. Conversely, in BD men, an attenuated and slower overall decrease of erBP during response preparation was partially mediated by impulsivity, which may contribute to their inability to refrain from risky behaviors. These findings suggest sex‐specific pathways of vulnerability in BDs. Overall, these results support the claim that binge drinking is associated with neural deficits resembling those seen in AUD, reflected in alterations of conflict processing and response execution.

## FUNDING INFORMATION

This work was supported by start‐up funds from the College of Sciences at San Diego State University and the National Institute on Alcohol Abuse and Alcoholism under award number R21AA027371 to K.M.

## CONFLICT OF INTEREST STATEMENT

All authors declare that they have no conflicts of interest.

## Supporting information


Appendix S1

## Data Availability

The data that support the findings of this study are available from the corresponding author upon reasonable request.

## References

[acer70252-bib-0001] Alderson Myers, A.B. , Arienzo, D. , Molnar, S.M. & Marinkovic, K. (2021) Local and network‐level dysregulation of error processing is associated with binge drinking. NeuroImage: Clinical, 32, 102879. Available from: 10.1016/j.nicl.2021.102879 34768146 PMC8591397

[acer70252-bib-0002] Almeida‐Antunes, N. , Crego, A. , Carbia, C. , Sousa, S.S. , Rodrigues, R. , Sampaio, A. et al. (2021) Electroencephalographic signatures of the binge drinking pattern during adolescence and young adulthood: a PRISMA‐driven systematic review. NeuroImage: Clinical, 29, 102537. Available from: 10.1016/j.nicl.2020.102537 33418172 PMC7803655

[acer70252-bib-0003] Barone, J. & Rossiter, H.E. (2021) Understanding the role of sensorimotor beta oscillations. Frontiers in Systems Neuroscience, 15, 655886. Available from: 10.3389/fnsys.2021.655886 34135739 PMC8200463

[acer70252-bib-0004] Barth, B. , Rohe, T. , Deppermann, S. , Fallgatter, A.J. & Ehlis, A.C. (2021) Neural oscillatory responses to performance monitoring differ between high‐ and low‐impulsive individuals, but are unaffected by TMS. Human Brain Mapping, 42(8), 2416–2433. Available from: 10.1002/hbm.25376 33605509 PMC8090766

[acer70252-bib-0005] Bartholow, B.D. , Fleming, K.A. , Wood, P.K. , Cowan, N. , Saults, J.S. , Altamirano, L. et al. (2018) Alcohol effects on response inhibition: variability across tasks and individuals. Experimental and Clinical Psychopharmacology, 26(3), 251–267. Available from: 10.1037/pha0000190 29863383 PMC5991490

[acer70252-bib-0006] Beaton, L.E. , Azma, S. & Marinkovic, K. (2018) When the brain changes its mind: oscillatory dynamics of conflict processing and response switching in a flanker task during alcohol challenge. PLoS One, 13(1), e0191200. Available from: 10.1371/journal.pone.0191200 29329355 PMC5766228

[acer70252-bib-0007] Bishop, F.M. & Rodriguez Orjuela, J.L. (2018) Toward the prevention of alcohol use disorders: overdrinking (unintentional binge drinking) in a community sample. Health Psychology Open, 5(2), 2,055,102,918,792,705. Available from: 10.1177/2055102918792705 PMC6108020 30151223

[acer70252-bib-0008] Carbia, C. , López‐Caneda, E. , Corral, M. & Cadaveira, F. (2018) A systematic review of neuropsychological studies involving young binge drinkers. Neuroscience and Biobehavioral Reviews, 90, 332–349. Available from: 10.1016/j.neubiorev.2018.04.013 29678643

[acer70252-bib-0009] Cavanagh, J.F. & Frank, M.J. (2014) Frontal theta as a mechanism for cognitive control. Trends in Cognitive Sciences, 18(8), 414–421. Available from: 10.1016/j.tics.2014.04.012 24835663 PMC4112145

[acer70252-bib-0011] Correas, A. , Cuesta, P. , Rosen, B.Q. , Maestú, F. & Marinkovic, K. (2021) Compensatory neuroadaptation to binge drinking: human evidence for allostasis. Addiction Biology, 26(3), e12960. Available from: 10.1111/adb.12960 32885571 PMC7930152

[acer70252-bib-0012] Correas, A. , López‐Caneda, E. , Beaton, L. , Rodríguez Holguín, S. , García‐Moreno, L. , Antón‐Toro, L. et al. (2019) Decreased event‐related theta power and phase‐synchrony in young binge drinkers during target detection: an anatomically‐constrained MEG approach. Journal of Psychopharmacology, 33(3), 335–346. Available from: 10.1177/0269881118805498 30355025 PMC6401286

[acer70252-bib-0013] Coutlee, C.G. , Politzer, C.S. , Hoyle, R.H. & Huettel, S.A. (2014) An abbreviated impulsiveness scale (ABIS) constructed through confirmatory factor analysis of the BIS‐11. Archives of Scientific Psychology, 2(1), 1–12. Available from: 10.1037/arc0000005 26258000 PMC4527550

[acer70252-bib-0014] Crego, A. , Cadaveira, F. , Parada, M. , Corral, M. , Caamaño‐Isorna, F. & Rodríguez Holguín, S. (2012) Increased amplitude of P3 event‐related potential in young binge drinkers. Alcohol, 46(5), 415–425. Available from: 10.1016/j.alcohol.2011.10.002 22459872

[acer70252-bib-0015] Delorme, A. & Makeig, S. (2004) EEGLAB: an open source toolbox for analysis of single‐trial EEG dynamics including independent component analysis. Journal of Neuroscience Methods, 134(1), 9–21. Available from: 10.1016/j.jneumeth.2003.10.009 15102499

[acer70252-bib-0016] Eysenck, S.B.G. , Eysenck, H.J. & Barrett, P. (1985) A revised version of the psychoticism scale. Personality and Individual Differences, 6(1), 21–29. Available from: 10.1016/0191-8869(85)90026-1

[acer70252-bib-0017] Fadardi, J.S. & Cox, W.M. (2006) Alcohol attentional bias: drinking salience or cognitive impairment? Psychopharmacology, 185(2), 169–178. Available from: 10.1007/s00213-005-0268-0 16491429

[acer70252-bib-0018] Hanslmayr, S. , Pastötter, B. , Bäuml, K.‐H. , Gruber, S. , Wimber, M. & Klimesch, W. (2008) The electrophysiological dynamics of interference during the Stroop task. Journal of Cognitive Neuroscience, 20(2), 215–225. Available from: 10.1162/jocn.2008.20020 18275330

[acer70252-bib-0019] Happer, J.P. , Beaton, L.E. , Wagner, L.C. , Hodgkinson, C.A. , Goldman, D. & Marinkovic, K. (2024) Neural indices of heritable impulsivity: impact of the COMT Val158Met polymorphism on frontal beta power during early motor preparation. Biological Psychology, 191, 108826. Available from: 10.1016/j.biopsycho.2024.108826 38862067 PMC11853962

[acer70252-bib-0020] Harper, J. , Malone, S.M. & Iacono, W.G. (2018a) Conflict‐related medial frontal theta as an endophenotype for alcohol use disorder. Biological Psychology, 139, 25–38. Available from: 10.1016/j.biopsycho.2018.10.002 30300674 PMC6299837

[acer70252-bib-0021] Harper, J. , Malone, S.M. & Iacono, W.G. (2018b) Impact of alcohol use on EEG dynamics of response inhibition: a cotwin control analysis. Addiction Biology, 23(1), 256–267. Available from: 10.1111/adb.12481 27859998 PMC5436950

[acer70252-bib-0022] Holcomb, L.A. , Huang, S. , Cruz, S.M. & Marinkovic, K. (2019) Neural oscillatory dynamics of inhibitory control in young adult binge drinkers. Biological Psychology, 146, 107732. Available from: 10.1016/j.biopsycho.2019.107732 31344371 PMC6718351

[acer70252-bib-0023] Huang, S. , White, D.R. & Marinkovic, K. (2022) Alterations of theta power and synchrony during encoding in young adult binge drinkers: subsequent memory effects associated with retrieval after 48 h and 6 months. Frontiers in Psychology, 13, 1061016. Available from: 10.3389/fpsyg.2022.1061016 36591031 PMC9798430

[acer70252-bib-0024] Koob, G.F. (2013) Theoretical frameworks and mechanistic aspects of alcohol addiction: alcohol addiction as a reward deficit disorder. Current Topics in Behavioral Neurosciences, 13, 3–30. Available from: 10.1007/7854_2011_129 21744309 PMC3448980

[acer70252-bib-0025] Koob, G.F. & Le Moal, M. (2008) Addiction and the brain antireward system. Annual Review of Psychology, 59(1), 29–53. Available from: 10.1146/annurev.psych.59.103006.093548 18154498

[acer70252-bib-0026] Koob, G.F. & Volkow, N.D. (2010) Neurocircuitry of addiction. Neuropsychopharmacology, 35(1), 217–238. Available from: 10.1038/npp.2009.110 19710631 PMC2805560

[acer70252-bib-0027] Kovacevic, S. , Azma, S. , Irimia, A. , Sherfey, J. , Halgren, E. & Marinkovic, K. (2012) Theta oscillations are sensitive to both early and late conflict processing stages: effects of alcohol intoxication. PLoS One, 7(8), e43957. Available from: 10.1371/journal.pone.0043957 22952823 PMC3428276

[acer70252-bib-0028] Kroenke, K. & Spitzer, R.L. (2002) The PHQ‐9: a new depression diagnostic and severity measure. Psychiatric Annals, 32(9), 509–515. Available from: 10.3928/0048-5713-20020901-06

[acer70252-bib-0029] Kuntsche, E. & Kuntsche, S. (2009) Development and validation of the drinking motive questionnaire revised short form (DMQ–R SF). Journal of Clinical Child & Adolescent Psychology, 38(6), 899–908. Available from: 10.1080/15374410903258967 20183672

[acer70252-bib-0030] Kwako, L.E. , Momenan, R. , Litten, R.Z. , Koob, G.F. & Goldman, D. (2016) Addictions neuroclinical assessment: a neuroscience‐based framework for addictive disorders. Biological Psychiatry, 80(3), 179–189. Available from: 10.1016/j.biopsych.2015.10.024 26772405 PMC4870153

[acer70252-bib-0032] Lange, J.E. & Voas, R.B. (2001) Defining binge drinking quantities through resulting blood alcohol concentrations. Psychology of Addictive Behaviors, 15(4), 310–316. Available from: 10.1037/0893-164X.15.4.310 11767262

[acer70252-bib-0033] Le Berre, A.P. , Fama, R. & Sullivan, E.V. (2017) Executive functions, memory, and social cognitive deficits and recovery in chronic alcoholism: a critical review to inform future research. Alcoholism, Clinical and Experimental Research, 41(8), 1432–1443. Available from: 10.1111/acer.13431 28618018 PMC5531758

[acer70252-bib-0035] Marinkovic, K. , Beaton, L.E. , Rosen, B.Q. , Happer, J.P. & Wagner, L.C. (2019) Disruption of frontal lobe neural synchrony during cognitive control by alcohol intoxication. Journal of Visualized Experiments, 144, e58839. Available from: 10.3791/58839 PMC6677147 30799848

[acer70252-bib-0036] Marinkovic, K. , Rickenbacher, E. , Azma, S. & Artsy, E. (2012) Acute alcohol intoxication impairs top–down regulation of Stroop incongruity as revealed by blood oxygen level‐dependent functional magnetic resonance imaging. Human Brain Mapping, 33(2), 319–333. Available from: 10.1002/hbm.21213 21391268 PMC3754428

[acer70252-bib-0037] Marinkovic, K. , Rosen, B.Q. , Cox, B. & Kovacevic, S. (2012) Event‐related theta power during lexical–semantic retrieval and decision conflict is modulated by alcohol intoxication: anatomically‐constrained MEG. Frontiers in Psychology, 3, 121. Available from: 10.3389/fpsyg.2012.00121 22536192 PMC3334511

[acer70252-bib-0038] Meyers, J.L. , Brislin, S.J. , Kamarajan, C. , Plawecki, M.H. , Chorlian, D. , Anohkin, A. et al. (2023) The collaborative study on the genetics of alcoholism: brain function. Genes, Brain and Behavior, 22(5), e12862. Available from: 10.1111/gbb.12862 37587903 PMC10550791

[acer70252-bib-0039] Mitchell, M.R. & Potenza, M.N. (2015) Importance of sex differences in impulse control and addictions. Frontiers in Psychiatry, 6, 22. Available from: 10.3389/fpsyt.2015.00024 25762943 PMC4332159

[acer70252-bib-0040] Molnar, S.M. , Beaton, L.E. , Happer, J.P. , Holcomb, L.A. , Huang, S. , Arienzo, D. et al. (2018) Behavioral and brain activity indices of cognitive control deficits in binge drinkers. Brain Sciences, 8(1), 1. Available from: 10.3390/brainsci8010009 PMC5789340 29300304

[acer70252-bib-0041] Murray, L. , Welsh, J.C. , Johnson, C.G. , Kaiser, R.H. , Farchione, T.J. & Janes, A.C. (2022) Alcohol‐ and non‐alcohol‐related interference: an fMRI study of treatment‐seeking adults with alcohol use disorder. Drug and Alcohol Dependence, 235, 109462. Available from: 10.1016/j.drugalcdep.2022.109462 35462263 PMC9106927

[acer70252-bib-0042] National Institute on Alcohol Abuse and Alcoholism . (2016) Drinking levels defined . https://www.niaaa.nih.gov/alcohol‐health/overview‐alcohol‐consumption/moderate‐binge‐drinking

[acer70252-bib-0043] Nixon, S.J. , Prather, R. & Lewis, B. (2014) Sex differences in alcohol‐related neurobehavioral consequences. Handbook of Clinical Neurology, 125, 253–272. Available from: 10.1016/B978-0-444-62619-6.00016-1 25307580

[acer70252-bib-0044] Norman, D.A. & Shallice, T. (1986) Attention to action: willed and automatic control of behavior. In: Davidson, R.J. , Schwartz, G.E. & Shapiro, D. (Eds.) Consciousness and self‐regulation: advances in research and theory Volume 4. Boston, MA: Springer US, pp. 1–18. Available from: 10.1007/978-1-4757-0629-1_1

[acer70252-bib-0045] Oostenveld, R. , Fries, P. , Maris, E. & Schoffelen, J.‐M. (2011) FieldTrip: open source software for advanced analysis of MEG, EEG, and invasive electrophysiological data. Computational Intelligence and Neuroscience, 2011(1), 156,869. Available from: 10.1155/2011/156869 PMC3021840 21253357

[acer70252-bib-0046] Oscar‐Berman, M. & Marinković, K. (2007) Alcohol: effects on neurobehavioral functions and the brain. Neuropsychology Review, 17(3), 239–257. Available from: 10.1007/s11065-007-9038-6 17874302 PMC4040959

[acer70252-bib-0047] Pandey, A.K. , Kamarajan, C. , Rangaswamy, M. & Porjesz, B. (2012) Event‐related oscillations in alcoholism research: a review. Journal of Addiction Research & Therapy, 7, 3844. Available from: 10.4172/2155-6105.S7-001 PMC3835599 24273686

[acer70252-bib-0048] Patrick, M.E. & Terry‐McElrath, Y.M. (2019) Prevalence of high‐intensity drinking from adolescence through young adulthood: national data from 2016–2017. Substance Abuse Research and Treatment, 13, 1178221818822976. Available from: 10.1177/1178221818822976 30718957 PMC6348505

[acer70252-bib-0049] Peter, J. , Ferraioli, F. , Mathew, D. , George, S. , Chan, C. , Alalade, T. et al. (2022) Movement‐related beta ERD and ERS abnormalities in neuropsychiatric disorders. Frontiers in Neuroscience, 16, 1045715. Available from: 10.3389/fnins.2022.1045715 36507340 PMC9726921

[acer70252-bib-0050] Rawlings, D. (1984) The correlation of EPQ psychoticism with two behavioural measures of impulsivity. Personality and Individual Differences, 5(5), 591–594. Available from: 10.1016/0191-8869(84)90034-5

[acer70252-bib-0051] Rosen, B.Q. , Padovan, N. & Marinkovic, K. (2016) Alcohol hits you when it is hard: intoxication, task difficulty, and theta brain oscillations. Alcoholism, Clinical and Experimental Research, 40(4), 743–752. Available from: 10.1111/acer.13014 27012442 PMC4820362

[acer70252-bib-0052] Ruiz, S.M. & Oscar‐Berman, M. (2013) Closing the gender gap: the case for gender‐specific alcoholism research. Journal of Alcoholism and Drug Dependence, 1(6), e106. Available from: 10.4172/2329-6488.1000e106 27500179 PMC4975372

[acer70252-bib-0053] Ryu, E. (2015) Multiple‐group analysis approach to testing group difference in indirect effects. Behavior Research Methods, 47(2), 484–493. Available from: 10.3758/s13428-014-0485-8 24907002

[acer70252-bib-0054] Schuckit, M.A. , Smith, T.L. & Tipp, J.E. (1997) The self‐rating of the effects of alcohol (SRE) form as a retrospective measure of the risk for alcoholism. Addiction, 92(8), 979–988. Available from: 10.1111/j.1360-0443.1997.tb02977.x 9376780

[acer70252-bib-0055] Schulte, T. , Müller‐Oehring, E.M. , Sullivan, E.V. & Pfefferbaum, A. (2012) Synchrony of corticostriatal‐midbrain activation enables normal inhibitory control and conflict processing in recovering alcoholic men. Biological Psychiatry, 71(3), 269–278. Available from: 10.1016/j.biopsych.2011.10.022 22137506 PMC3253929

[acer70252-bib-0056] Selzer, M.L. , Vinokur, A. & Van Rooijen, L. (1975) A self‐administered Short Michigan Alcoholism Screening Test (SMAST). Journal of Studies on Alcohol, 36(1), 117–126. Available from: 10.15288/jsa.1975.36.117 238068

[acer70252-bib-0555] Sobell, L. C. , & Sobell, M.B. (1992) Timeline follow‐back: A technique for assessing self‐reported alcohol consumption. In: Litten, R.Z. , Allen, J.P. (Eds.) Measuring Alcohol Consumption, Totowa, NJ: Humana Press, 10.1007/978-1-4612-0357-5_3

[acer70252-bib-0057] Spitzer, R.L. , Kroenke, K. , Williams, J.B.W. & Löwe, B. (2006) A brief measure for assessing generalized anxiety disorder: the GAD‐7. Archives of Internal Medicine, 166(10), 1092–1097. Available from: 10.1001/archinte.166.10.1092 16717171

[acer70252-bib-0058] Tzagarakis, C. , Thompson, A. , Rogers, R.D. & Pellizzer, G. (2019) The degree of modulation of beta band activity during motor planning is related to trait impulsivity. Frontiers in Integrative Neuroscience, 13, 1. Available from: 10.3389/fnint.2019.00001 30705624 PMC6344424

[acer70252-bib-0059] Weafer, J. (2020) Sex differences in neural correlates of inhibitory control. In: de Wit, H. & Jentsch, J.D. (Eds.) Recent advances in research on impulsivity and impulsive behaviors. Current topics in behavioral neurosciences, Vol. 47. Cham: Springer. Available from: 10.1007/7854_2020_146 32462613

[acer70252-bib-0060] Weintraub, S. , Dikmen, S.S. , Heaton, R.K. , Tulsky, D.S. , Zelazo, P.D. , Bauer, P.J. et al. (2013) Cognition assessment using the NIH toolbox. Neurology, 80(11), S54–S64. Available from: 10.1212/WNL.0b013e3182872ded 23479546 PMC3662346

[acer70252-bib-0061] White, A.M. (2020) Gender differences in the epidemiology of alcohol use and related harms in the United States. Alcohol Research: Current Reviews, 40, 1. Available from: 10.35946/arcr.v40.2.01 PMC7590834 33133878

[acer70252-bib-0062] Wilcox, C.E. , Dekonenko, C.J. , Mayer, A.R. , Bogenschutz, M.P. & Turner, J.A. (2014) Cognitive control in alcohol use disorder: deficits and clinical relevance. Reviews in the Neurosciences, 25(1), 1–24. Available from: 10.1515/revneuro-2013-0054 24361772 PMC4199648

