# Supplementary material for: Oscillatory and behavioral indices of cognitive control dysregulation in young adult binge drinkers are influenced by sex differences
Source: Alcohol Clin Exp Res (Hoboken). 2026 Feb 16;50(2):e70252. doi: 10.1111/acer.70252 (PMC12910152; doi:10.1111/acer.70252)
Supplement: Supplementary file 1 — Appendix S1 [file ACER-50-0-s001.docx]

Supplementary material

**Table 2**

Parameter estimates for the multiple-group mediation model of group (BD vs. LD) effects on reaction times via event-related theta power (erTP) in the high conflict, incongruous–congruous (I-C) contrast condition for women and men.

|  | **Women** | | | **Men** | | |
| --- | --- | --- | --- | --- | --- | --- |
| **Parameter** | **Est.** | ***p*** | ***95% CI*** | **Est.** | ***p*** | ***95% CI*** |
| a | .76 | .03 | [0.10; 1.44] | .20 | .53 | [-0.40; 0.82] |
| b | -.40 | .01 | [-0.71; -0.14] | -.24 | .17 | [-0.55; 0.15] |
| c‘ | .00 | .99 | [-0.69; 0.73] | -.48 | .18 | [-1.17; 0.24] |
| ab | -.30 | .08 | [-0.70; -0.02] | -.05 | .62 | [-0.29; 0.11] |
| (ab)+c‘ | -.30 | .35 | [-0.93; 0.36] | -.53 | .14 | [-1.22; 0.20] |

**Table 3**

Parameter estimates for the multiple-group mediation model of group (BD vs. LD) effects on beta power via impulsivity for women and men.

|  | **Women** | | | **Men** | | |
| --- | --- | --- | --- | --- | --- | --- |
| **Parameter** | **Est.** | ***p*** | ***95% CI*** | **Est.** | ***p*** | ***95% CI*** |
| a | -.22 | .001 | [-.35; -.09] | -.27 | .002 | [-.45; -.10] |
| b | -.01 | .77 | [-.05; .03] | .13 | <.001 | [.07; .20] |
| c‘ | -.01 | .82 | [-.05; .04] | -.08 | .004 | [-.14; -.03] |
| ab | .002 | .79 | [-.01; .02] | -.04 | .04 | [-.08; -.01] |
| (ab)+c‘ | -.003 | .90 | [-.05; .04] | -.12 | <.001 | [-.18; -.06] |
